# Platelet- Rich Plasma Treatment Supported by Ultrasound Detection of Septa in Recurrent Canine Aural Hematoma: A Case Series

**DOI:** 10.3390/ani13152456

**Published:** 2023-07-29

**Authors:** Paola Palagiano, Lisa Graziano, Walter Scarabello, Priscilla Berni, Valentina Andreoli, Stefano Grolli

**Affiliations:** 1 Clinica Veterinaria Meda, 20821 Meda, Italy; p.palagiano@cvmeda.it (P.P.); l.graziano@cvmeda.it (L.G.); 2 MYPETCLINIC s.r.l., 20149 Milano, Italy; walterscarabello@hotmail.com; 3 Dipartimento di Scienze Medico-Veterinarie, Università degli Studi di Parma, 43126 Parma, Italy; priscilla.berni@unipr.it (P.B.); stefano.grolli@unipr.it (S.G.)

**Keywords:** aural hematoma, platelet-rich plasma, canine, treatment, regenerative therapies

## Abstract

**Simple Summary:**

Canine aural hematoma management usually involves invasive surgery or prolonged pharmacologic treatments and is often linked to a high relapse rate. A correct diagnosis, associated with proper follow-up, is crucial for the success of the treatment. In this work, we report the treatment of 11 dogs (12 ears) affected by both acute and chronic aural hematoma with an innovative therapy based on Platelet- Rich Plasma (PRP). Ultrasound guidance was used to identify the presence of multiple and non-communicating chambers. Furthermore, in patients with pendulous ears, a bandage was applied to facilitate the post-treatment management. The results obtained in the study suggest that the PRP injection can be an effective alternative for the treatment of both chronic and acute aural hematoma without relapses in the long-term follow-up.

**Abstract:**

Aural hematoma is a common pathological condition in veterinary practice with a high incidence rate in dogs. Drainage, corticosteroid injections, and surgical approaches represent the common treatments in these clinical cases. However, surgery leaves visible signs and is usually correlated with recurrence, scars, and deformation of the treated pinna. For this reason, more effective and less invasive methods have been proposed over the years. Platelet-Rich Plasma (PRP) is one of the most promising options due to its pro-regenerative properties and capability to modulate the inflammatory state. The present work reports 12 cases of canine aural hematoma treated with PRP. The PRP treatment was combined with an ultrasound evaluation of the pinna to detect and treat all involved septa. The results show that relatively large volumes (2 mL) of PRP associated with an ultrasound guide are safe and efficacious in the treatment of canine aural hematoma requiring a maximum of two infiltrations, both in acute and chronic conditions. All the patients recovered their normal ear thickness (compared with the controlateral one) without relapses, averaging 38.5 days from their first treatment (10–90 days; SD: 24.7). The key role of PRP combined with a tailored diagnosis process carried out by the veterinarian, which included using an ultrasound system and the proper bandage, suggests that this approach may represent a valid alternative to surgery and corticosteroids.

## 1. Introduction

Aural hematoma is a disease characterized by the spilling of serosanguinous fluid inside the pinna due to traumatic rupture of the capillaries [1,2,3]. Although the disease is common in both cats and dogs, the latter are more likely to develop aural hematoma, especially breeds with pendulous ears. Aural hematomas cause pain and discomfort due to swelling and inflammation of the pinna and the weight of the affected area [4]. The pathogenesis of aural hematoma is partially unclear. However, among the leading causes of blood accumulation are ear infections, such as otitis, ear parasitosis, immunogenic diseases, or, more simply, excessive ear shaking [2,3,5,6,7]. Determining and treating the underlying cause of the aural hematoma is critical for a clinical treatment’s success. In the case of otitis, this should be treated accordingly. Chronic aural hematoma can lead to chronic inner organization in the pinna, resulting in fibrosis, increasing the predisposition of the patient to subsequent ear infections, as well as severe aesthetic morphological distortion of the affected pinna (‘cauliflower ear’) [4,8,9]. The most used therapeutic approach is to drain excess blood [3,10] and immediately inject drugs such as corticosteroids. Steroids have, however, unclear and highly variable results, with a high risk of recurrence [3,11,12,13]. Surgery is the second most common approach. The procedure consists of an incision along the long axis of the pinna, drainage of the hematoma, and application of lateral sutures to avoid skin detachment from the auricular cartilage [3,9]. Since the pathogenesis is doubtful, even in the case of surgery, there are conflicting results and complications such as incomplete healing, infections, necrosis, thickening, and wrinkling of the auricle [9,14]. Surgery is also an invasive approach, requiring sedation of the patient. A suitable treatment for aural hematoma should avoid recurrence, be non-invasive, and shorten the healing time [11,15]. For all the reasons listed above, an accurate diagnosis and alternative non-invasive approaches are essential to reach clinical efficacy and reduce the risk of recurrences.

Platelet- Rich Plasma (PRP) is a blood-derived product characterized by a high concentration of platelets and growth factors [16,17,18] and studied for its immunomodulatory properties and ability to support the healing process in various pathologies [19,20,21]. PRP is usually obtained via a double centrifugation process of a sample of whole peripheral blood collected in the presence of anticoagulants [22]. This autologous biological product represents a natural mix of active metabolites and growth factors that synergistically contribute to activating regenerative physiological pathways to resolve persistent pathological states [23]. To be effective with a significant clinical outcome, PRP must have a minimum platelet concentration, quantified as a 3×–5× (3–5-fold increase in platelet concentration compared with whole blood) [24,25,26]. The release of growth factors and their concentration in PRP is strictly related to platelet concentration. Growth factors that play a critical role in coordinating the tissue regeneration process are transforming growth factor β (TGF-β), platelet-derived growth factor (PDGF), fibroblast growth factor (FGF), epidermal growth factor (EGF), insulin-like growth factor (IGF), and vascular endothelial growth factor (VEGF) [27,28,29], although activated platelets secrete several other bioactive molecules. 

The methodology and procedure to obtain PRP from a whole blood sample are crucial to determine the product’s clinical efficacy. PRP can be prepared using a manual process or through a commercial kit. The manual process is an open technique and requires using a biological hood to avoid contaminations and risks of infections and good manual capability and experience to work in sterility. Commercial kits offer simpler, closed, guided systems usually based on single or double centrifugation of whole blood. These systems are sometimes associated with low efficiency of concentrating platelets, resulting in non-repeatable and variable results [30,31,32].

Considering its capabilities to induce neo-angiogenesis, activate regenerative pathways, enhance the deposition of the extracellular matrix, and modulate the inflammatory response and its bacteriostatic power, PRP represents an attractive minimally invasive therapeutic option for the treatment of aural hematoma [33].

However, many aspects can influence the clinical outcome in PRP treatment of aural hematoma. It is essential to consider, indeed, the diagnostic process, the volume of PRP used, the type of bandage, and the follow-up management. 

The present study aimed to assess the safety and clinical efficacy of a novel protocol for the treatment of aural hematoma based on the use of diagnostic imaging and local administration of PRP. Furthermore, in patients with pendulous ears, the PRP application was followed by a specific bandage procedure. The protocol was applied in an 11-patient trial (12 ears). To our knowledge, a clinical protocol for the PRP treatment of canine aural hematoma based on diagnostic ultrasound imaging and direct application of PRP has not been described before. 

## 2. Materials and Methods

### 2.1. Ethics Statement

Eleven client-owned dogs were enrolled in the study, and written informed consent was obtained from all pet owners involved. The study was conducted in accordance with the principles of Good Clinical Practice.

### 2.2. Animals and Inclusion Criteria

Eleven dogs of different breeds and ages affected by recurrent unilateral aural hematoma were included in the study. All the enrolled cases were previously treated with traditional needle drainages without resolution and with hematoma recurrence. The inclusion criteria were the presence of aural hematoma associated with pruritus, shaking of the head, and normal routine blood analysis. At the time of enrollment, the authors considered an aural hematoma to be acute if it occurred within the past 5 days, and chronic if it had been present for more than 5 days.

A complete clinical and cytological check was performed to diagnose underlying otitis. If present, the otitis was treated with local application of Osurnia^®^, Dechra, or Neptra^®^ (Bayer Animal Health GmbH, Leverkusen, Germany). During the study, pictures of the affected ear and information about the patient’s clinical state were recorded.

Out of the twelve ears treated in the study, ten were acute, while two were chronic aural hematoma. Patient 1 had an aural hematoma on the left ear (1L), then a new aural hematoma occurred in the controlateral pinna (1R) after a few months and was treated with the same protocol. One chronic patient had a recurrent aural hematoma already treated with medical and surgical intervention without success and with further recurrence of the swelling. The other chronic patient had an aural hematoma lasting more than 30 days with a scar already present.

All patients included in the study presented single or two communicating- chamber hematoma, except for the last one. In detail, patient 11 was an 8-year-old female Amstaff affected by Leishmania, diagnosed with aural hematoma caused by Malassezia otitis. The hematoma was diagnosed 15 days before PRP treatment to the right ear. The ultrasound detection revealed the presence of numerous fibrous septa, which created communicating chambers. However, these partitions also created 2 large non-communicating chambers that were drained and infiltrated separately. The patient’s ear was bandaged, and the same treatment was repeated on the seventh day. 

### 2.3. Autologous Platelet- Rich Plasma Preparation

Autologous Platelet-Rich Plasma was prepared through a semi-automated closed and sterilized disposable kit (Ematik^®^ Kit Semi-manuale, Prometheus Srl, Parma, PR, Italy) at the moment of enrollment (D0). Briefly, the kit included the equipment for (i) the blood collection, from a minimum of 18 to a maximum of 55 mL of blood volume, with the addition of anticoagulant; (ii) the PRP preparation, using a closed system of tubes, without the need for a biological hood; (iii) the PRP application. The kit was also supplied with a device for better handling and more efficient separation of the plasma layer from red blood cells after the first centrifugation step. The kit needed a non-proprietary centrifuge with baskets having at least a 175 mL inner volume (4 × 175 mL swing out, Neya 8 Basic). The blood withdrawing kit included a 20 G butterfly needle, a 60 mL syringe, a 4% trisodium citrate prefilled syringe (5 mL) as anticoagulant, and a 60 mL medical bag for the first centrifugation of the whole blood (WB). A total of 5 mL of anticoagulant was added to the 60 mL syringe containing 55 mL of whole blood collected on each patient. The anticoagulated blood was transferred to the medical bag and centrifuged with proprietary protocols.

**Figure 1:**
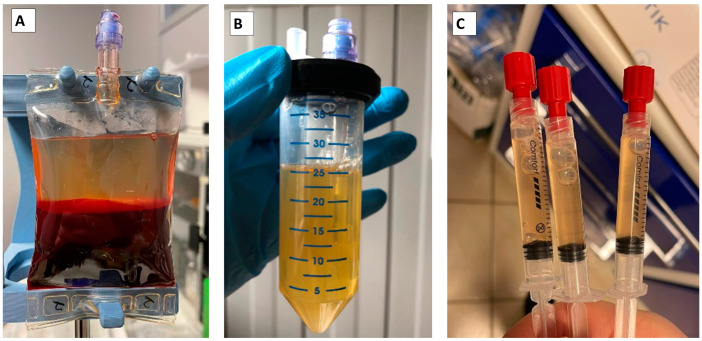
The picture shows the kit bag containing red blood cells separated from the plasma layer after the first centrifugation step (**A**); plasma recovered and placed in the conical container for the second centrifugation (**B**); three doses of 2 mL each of PRP (**C**).

After the first centrifugation performed at 800× *g* for 7 min, the three layers of red blood cells, buffy coat, and plasma-containing platelets were clearly visible, with no or negligible contamination of the plasma layer with red blood cells (Figure 1A).

The medical bag was connected to the line of the kit’s tubes for the separation of the plasma layer from red blood cells. The tubes’ line connected, in a closed system, the blood bag to a conical container which was needed for the second centrifugation of plasma (Figure 1B). Using a syringe included in the closed system, the plasma layer was transferred from the bag into the conical container.

The plasma in the conical container underwent a second centrifugation at 900× *g* for 15 min. The second centrifugation was needed to concentrate platelets. Following the second spin, the plasma sample was divided into a supernatant composed of Platelet- Poor Plasma (PPP) and a pellet of platelets. The large part of PPP was removed through a sterile syringe connected to a swab-able connector of the conical container, leaving only 6 mL of PPP inside the conical container. The PPP was then mixed with the platelet pellet using a sterile syringe connected to the swab-able connector of the conical container and performing an “up and down” movement of the piston. This fluid movement allowed us to suspend homogenous platelets in a closed system, creating a solution enriched in platelets and without aggregates. The final PRP solution was then collected and separated into 3 syringes of 2 mL each to obtain 3 doses of PRP (Figure 1C). 

One dose was applied as fresh PRP solution and the other 2 were frozen at −20 °C up to 30 days for further possible treatments. 

Whole blood and PRP samples were analyzed with a blood-cell counter (Cell-Dyn 3500 CS, Abbott, Abbott Park, IL, USA) to establish the amount of platelets retrieved from each patient. Data coming from the counts were used to define PRP’s quality (Table 1).

### 2.4. PRP Treatments

All patients were treated without undergoing sedation or anesthesia. The ear of each patient was disinfected, and hair was removed. The ultrasound analysis was performed using a Mindray M9 ecograph and a sterile linear probe. The ultrasound probe was placed into the concave surface of the pinna. Minor adjustments of probe positioning were made to provide an adequate ultrasound signal of the whole area. The ultrasound signal was displayed on a monitor screen. After an appropriate signal acquisition, the presence of communicating or non-communicating chambers was detected, and a pair of 20 G butterfly needles per ear chamber was inserted in correspondence of the hematoma. One needle was used for the drainage of the hematoma, and the other one was for the infiltration of PRP. The latter was pre-filled with PRP to avoid inserting air inside the pinna during the infiltration. 

Both butterflies had to be inserted before performing drainage since, after the hematoma has been drained, the pinna collapses, making it impossible to insert the second butterfly for PRP infiltration in the cavity. 

Depending on the shape of the ear, the needles were inserted in the upper part or at the base of the ear for straight or pendulous ears, respectively. This procedure was aimed at avoiding crumpling or excessive pressure on the injured site.

Briefly, the ear was disinfected, the first butterfly was inserted in the hematoma chamber and connected to a sterile 30 mL syringe (or more than one depending on the volume to be drained) to drain the hematoma, and the second pre-filled butterfly connected to the PRP syringe was inserted before performing the drainage. The drained volume of blood was registered. After the first needle removal, 2 mL of autologous PRP were instilled. At the end of the procedure, the second needle was also removed, and the area was disinfected and massaged with gentle pressure for 1–2 min. 

Furthermore, a compressive bandage was applied and maintained for at least 72 h in 4 of the patients enrolled. In particular, the bandage was applied only in animals with pendulous ears to provide support and avoid damage to the pinna during head shaking and scratching. In detail, the bandaging procedure consisted of a gauze curler placed on the concave surface of the pinna. Then, the ear was folded inwards, forming a sort of flap, and fixed with two turns of bandage around the head: the first in cotton and the second in a hemmed bandage. Everything was fixed with adhesive surgical tape. A bandage tutorial is available in the Supplementary Materials, File S1. An Elizabethan collar was worn for 3 days only when dogs attempted to scratch the lesion. A recheck evaluation was performed 72 h post-treatment to evaluate the lesion and provide permission for removal of the collar.

A second treatment was repeated within 30 days if the ear swelling was equal to or increased compared with that at D0. In detail: if, during the check on Days 7, 15, and 30 the ear swelling measured with the caliper was equal to or higher than the one present on Day 0, a second treatment was performed repeating the same procedures. If the caliper measure was less than the one present on Day 0, no additional treatments were applied.

### 2.5. Clinical Evaluation

The patients were examined once a week until complete healing, defined as the recovery of the normal ear thickness compared with the controlateral one, associated with the absence of pruritus and head shaking. 

At each time point, ear thickness was measured, pictures were taken, and possible scarring, contractures, and deformations were assessed. 

## 3. Results

### 3.1. Quality of PRP

The PRP obtained with the Ematik^®^ kit had an average PLT (platelet) concentration of 1.18 × 10^6^/μL with a standard deviation (SD) of 6.07 × 10^5^ PLT/μL (maximum value: 1.92 × 10^6^/μL, minimum value: 3.29 × 10^5^/μL), with a 51.5% mean platelet recovery from the whole blood (SD: 21%) and a mean PLT concentration factor of 5× (SD: 2.2×).

The recovery of white blood cells had an average of 3.5% from whole blood (SD: 1.6%) and an average of 1% of red blood cells (SD: 0.5%). These data are shown in Table 1. 

### 3.2. Clinical Study Results 

At D0, the mean volume drained from the patients was 18.5 mL (8–40 mL; SD: 10.3).

All the aural hematomas were treated with PRP infiltrations, and a total of eighteen treatments were performed. 

In detail, six aural hematomas required only one PRP treatment, while six patients required a total of two PRP infiltrations, where the second one was carried out at D7 or D30. The average time for restoring the normal ear thickness compared with the controlateral ear was 38.5 days (10–90 days; SD: 24.7).

In the next few days after the first treatment, a non-painful swelling of the treated area equal to or smaller than the initial hematoma size was recorded in each patient. The swelling resolved or reduced in 10–30 days without any further treatment. Mild thickening of the pinna was registered in one dog. No deformations of the pinna or crumpling were registered.

No side effects or complications were recorded for any patient and no erythema, pain, or inflammation were registered after the PRP treatment. At the time of writing, the follow-up period ranged from 10 to 20 months, with a mean value of 14.58 months (SD: 3.7). Data relating to each case are shown in Table 2. 

**Figure 2:**
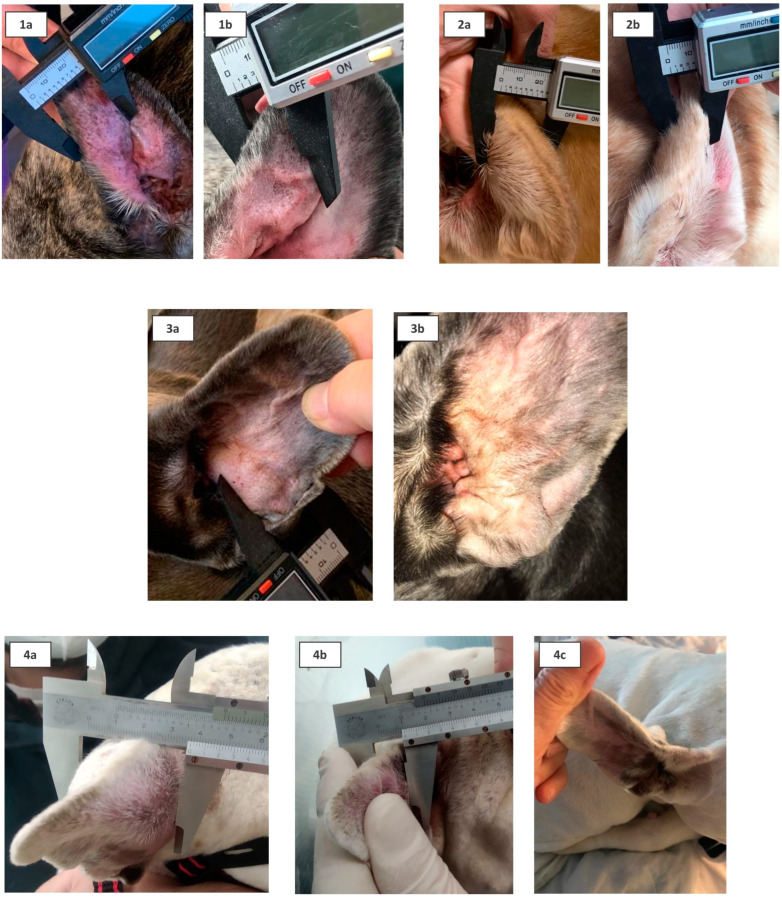
Representative pictures of the pinna of four patients (1 = case 1; 2 = case 3; 3 = case 5; 4 = case 11). In each case, the ear thickness was measured with a caliper before (letter a) and after (letters b and c) the PRP treatment. The swelling was decreased, and the ear came back to its natural thickness without scars.

Figure 2 shows the ear affected by aural hematoma of four patients, before and after PRP treatment. 

## 4. Discussion

Aural hematoma is characterized by a blood collection between the skin and the auricular cartilage of the pinna. It is one of the most frequent pathologies in dogs and cats, and the therapeutic approach is challenging and requires solving the triggering cause and removing the extravasation of blood to prevent possible recurrences [3,34]. Surgery is often an election technique for the treatment of aural hematoma. Several active and passive surgical drainages have been described in the literature, but all the approaches are invasive and involve the sedation of the animal, and often leave visible signs, causing discomfort for both dogs and owners [9,14,35,36]. The second most popular approach is the local or systemic application of corticosteroids [10,12,37]. Unfortunately, these treatments do not give repeatable results and often expose animals to recurrences or incomplete recovery [3,11,12,13]. Recently, some studies evaluated the possibility of a surgically applied vacuum drain with minimal cutting and without bandages [8,15]. The aesthetic results are excellent, but this technique involves animals’ sedation (often both during positioning and removal of drainage) and the use of an Elizabethan collar for the entire period of drainage maintenance, which may last from two to three weeks. 

The present study aimed to identify an alternative treatment for canine aural hematoma based on the application of PRP, avoiding surgery or invasive approaches, and allowing a permanent resolution of the inflammatory state. The clinical application of PRP in various pathologies has been extensively described as a promising therapy in the literature, based on its pro-regenerative, anti-inflammatory, and immunomodulatory properties [38,39]. Furthermore, some studies underline the marked antibacterial activity of this blood-derived product [40,41,42]. These characteristics suggest PRP as a candidate for the treatment of aural hematoma, especially considering that the effusion in the pinna can arise both from simple trauma (such as excessive shaking of the ears) or infection [5]. 

PRP preparation procedures vary considerably, and the heterogeneity of the characteristics and composition of the final products represent a severe obstacle to the replication of the therapeutic procedure and the understanding of the clinical effects [43]. Different methods have been proposed to classify the preparation of PRP and provide information about its application [24,30,44,45]. The characterization of the PRP used in this study and the procedure of preparation allowed us to define the obtained product as “pure-PRP” according to the classification by Ehrenfest et al. [24]. In fact, the obtained PRP had a platelet concentration between three and five times the concentration of the initial blood sample, and the level of residual white blood cells was less than 5% compared with whole blood. Furthermore, according to the PAW classification proposed by De Long et al. [44], the PRP used in the present study is classified P3-B, while following the DEPA classification [30], it is scored CCB. Supplementary File S2 provides more information about PRP classification and characterization.

Regarding the clinical applications, there is not much information about the use of blood products for the treatment of aural hematoma. In 2021, Perego and colleagues presented a case report describing the application of leukocyte-rich PRP (L-PRP) on 17 dogs affected by aural hematoma [33]. The absence of any reported side effects and the positive outcome of all treated subjects suggested the safety and efficacy of the therapy [33]. This early evidence supports the choice of a PRP-based protocol for the treatment of aural hematoma, which, in our work, has been combined with proper diagnosis, drainage, and ear bandage.

In 2006, Benigni and Lamb defined different types of diagnostic imaging of ear pathologies, including aural hematoma, underlining the importance of their use [46]. However, no combination of diagnostic imaging with PRP treatment has been described in the literature yet. In the present study, the support of ultrasound imaging before the PRP injection allowed for the correct identification of single or multiple chambers inside the pinna. Detecting the presence of fibrous septa and possible connections between them was crucial to drain and treat every affected part of the aural hematoma cavity. The use of this method, for example, was fundamental for treating every affected chamber in Case 1: in the right ear, as reported in Table 2, the patient presented an aural hematoma with a communicating double chamber. If the chambers had not been communicating, treating the entire affected area would not have been possible. Patient 11 highlights the importance of the ultrasound diagnostic method. In fact, without the ultrasound support, the presence of the two non-communicating chambers would not have been detected, and the patient would have received partial or inadequate treatment for the aural hematoma. 

A key point in the treatment of aural hematoma is drainage, as supported by some papers that highlight how this procedure, when performed correctly, can be particularly helpful in the initial phase of patient treatment [2,3,10]. In the present study, the drained volumes collected before the PRP injection were recorded. Interestingly, there seems to be a correlation between the drained volumes and the days of healing, suggesting that the greater the volume drained, the shorter the healing time, although further investigations are needed to confirm this hypothesis. Not only the volumes of blood drained, but also the volumes of PRP injected are crucial when considering the treatment of aural hematoma. In 2021, Perego et al. evaluated the in vivo clinical effect of 0.5–1 mL instillation of autologous L-PRP (autologous PRP and Leukocytes) in treating canine aural hematoma. The paper provides data in support of the critical activity carried out by the PRP, even in small doses in such a complex clinical framework [33,47]. In the present study, we have evaluated larger dosages, reaching 2 mL of PRP for both single and multiple applications. A single administration of pure-PRP was sufficient in the 50% of the treated ears, while others required a second administration. None of the treated cases needed a third PRP treatment. Additionally, none of the patients showed any side effects after the treatment, confirming the results of Perego and colleagues regarding the safety of this biological product [33], even when using larger volumes. 

The ear bandage is a challenging aspect of aural hematoma treatment. Lahiani and Niebauer reported worsening of otitis, the occurrence of infections, and tissue necrosis due to incorrect application of the dressing [15]. In this study, the PRP application was associated with a bandage only if the patients presented pendulous ears to facilitate post-treatment management. The bandage was designed to be well-tolerated by the dogs and to prevent further fluid accumulation in the pinna.

Ultimately, effective patient follow-up management is crucial to prevent recurrences. In the initial hours and days after the PRP infiltration, ear swelling could still be present, but continuous drainage, which could stimulate further bleeding from the damaged blood vessels, was avoided. If the swelling was still present during the weekly clinical check (Days 7, 15, 30), a second treatment was repeated. All the treatments were successful within 90 days of follow-up, with a mean healing time of 38.5 ± 24.7. The treatment carried out following the surgical approach involves the removal of stitches within ten days. However, the patient must undergo anesthesia, the method is invasive and painful, and the final result strictly depends on the owner’s actions. Moreover, it is reported that the average number of recurrences after surgery is about 25% [15]. In our study, the average healing time was longer than the post-surgery management, but it allowed for a less invasive treatment of the patient, who did not need anesthesia. Based on the experience reported in this work, it also enabled the resolution of aural hematoma without visible scarring or recurrences in the short and long term. Ten hematomas out of twelve were diagnosed as acute (83.3%), while two were chronic (16.7%). It is important to note that the two chronic animals presented pre-existing scars on the affected ears upon arrival due to a previous treatment with the surgical approach.

The authors are aware that, in the present study, the intralesional administration of PRP was not compared with a control group. This is a clear weakness of the work. In the future, a more complete clinical trial is needed to include a control group.

## 5. Conclusions

In conclusion, this case series shows that the local infiltration of relatively large volumes of PRP is safe and represents a promising therapy for recurrent canine aural hematoma. In the present study, the PRP treatment associated with a proper ultrasound diagnosis led to a long-term healing of the disease, without aesthetic alterations or deformations of the pinna.

## Figures and Tables

**Table 1:** The table shows the mean concentration values of WBCs (white blood cells), RBCs (red blood cells), and PLTs (platelets) in the whole blood and the PRP characteristics regarding cellular concentration and percentage of recovery with respect to the initial blood sample. A statistically significant difference was found between the whole blood and the PRP regarding WBC, RBC, and PLT concentrations (* means a *p* value < 0.001).

	Whole Blood Concentration	PRP Concentration ± SD	Concentration Factor	% Recovery	*p* Value
WBC	7.56 × 10^3^/μL	2.60 × 10^3^/μL ± 1.28 × 10^3^/μL	0.34	3.5%	*
RBC	6.12 × 10^6^/μL	4.98 × 10^5^/μL ± 3.31 × 10^5^/μL	0.08	1.0%	*
PLT	2.57 × 10^5^/μL	1.18 × 10^6^/μL ± 6.07 × 10^5^/μL	5	51.5%	*

**Table 2:** Clinical case resume regarding hematoma characteristics, PRP treatment, and follow-up. Case 4, marked with *, had a lower drained volume at D0 because drainage was performed in an emergency 24 h before the PRP treatment, but was not recorded.

Dog	Type	Drained mL D0	Previous Scars D0	Scars Related to PRP Treatment	N° of Non- Communicating Chambers	N° of Communicating Chambers	PRP Infiltrations	mL of PRP	Bandage	Days for Healing	Follow-Up (Months)
CASE 1L	Acute	12	NO	NO	0	1	2	2	NO	40	20
CASE 1R	Acute	20	NO	NO	0	2	1	2	NO	20	14
CASE 2	Acute	20	NO	NO	0	1	1	2	NO	37	19
CASE 3	Acute	14	NO	NO	0	1	1	2	YES	10	19
CASE 4	Chronic	8 *	YES	NO	0	1	1	2	NO	14	18
CASE 5	Acute	25	NO	NO	0	1	2	2	YES	60	14
CASE 6	Acute	35	NO	NO	0	1	1	2	NO	30	12
CASE 7	Acute	18	NO	NO	0	1	1	2	NO	30	12
CASE 8	Acute	10	NO	NO	0	1	2	2	NO	60	11
CASE 9	Acute	10	YES	NO	0	1	2	2	NO	60	10
CASE 10	Chronic	10	YES	NO	0	1	2	2	YES	90	10
CASE 11	Acute	40	NO	NO	2	3	2	2	YES	11	16
MEAN		18.5					1.5			38.5	14.58
ST.DEV.		10.3					0.52			24.7	3.70

## Data Availability

Raw data supporting the conclusions of this article will be made available by the authors.

## Supplementary Materials

The following supporting information can be downloaded at: https://www.mdpi.com/article/10.3390/ani13152456/s1. File S1: Bandage tutorial. File S2: PRP Classification. References [24,30,43,44,45] are cited in the supplementary materials.
