# Supplementary material for: Platelet- Rich Plasma Treatment Supported by Ultrasound Detection of Septa in Recurrent Canine Aural Hematoma: A Case Series
Source: Animals (Basel). 2023 Jul 29;13(15):2456. doi: 10.3390/ani13152456 (PMC10417061; doi:10.3390/ani13152456)
Supplement: Supplementary file 1 [file animals-13-02456-s001.zip › Supplementary material File S1.pdf]

SUPPLEMENTARY MATERIALS: FILE S1

Tutorial video explaining the ear bandage technique used in the present study: https://youtu.be/CLv8FJnkEl4

From: https://www.vetnursing.ie/ available online, last access on 20/07/2023
