# Supplementary material for: Platelet- Rich Plasma Treatment Supported by Ultrasound Detection of Septa in Recurrent Canine Aural Hematoma: A Case Series
Source: Animals (Basel). 2023 Jul 29;13(15):2456. doi: 10.3390/ani13152456 (PMC10417061; doi:10.3390/ani13152456)
Supplement: Supplementary file 1 [file animals-13-02456-s001.zip › Supplementary material File S2.pdf]

Classification of PRP used in the therapy of aural hematoma

	Whole Blood Concentration	PRP Concentration ± SD	Concentration factor	% Recovery	*p value*
WBC	7,56 x10^3/µL	2,60 x10^3/µL ± 1,28 x10^3/µL	0,34	3,5%	*
RBC	6,12 x10^6/µL	4,98 x10^5/µL ± 3,31 x10^5/µL	0,08	1,0%	*
PLT	2,57x10^5/µL	1,18x10^6 /µL ± 6,07 x10^5/µL	5	51,5%	*

The table illustrates the main characteristics of PRP used in the study in term of cell components. The mean concentration values are reported for WBC (white blood cells), RBC (red blood cells), and PLT (platelets) in the whole blood. Furthermore, the PRP characteristics regarding cellular concentration and percentage of recovery with respect to the initial blood sample are reported. A statistically significant difference was found between the whole blood and the PRP regarding WBC, RBC, and PLT concentrations (*means a p value <0,001).

Different classification systems have been proposed to characterize and classify PRP (Rossi et al., 2019).

To provide a description of the PRP used in the study the following classification methods have been evaluated.

1. **Dohan Ehrenfest et al., 2009**

   The classification is based on the recovery of platelets, the presence or absence of PRP, the eventual activation procedure.
   PRP characteristics:
   Platelet collection efficiency: good (platelet recovery between 40 and 80%)
   Leucocyte collection efficiency: no leucocytes (technique eliminates >95% leucocytes)
   No activating substances were used.
   The final definition of the PRP is: Pure-PRP

2. **De Long et al., 2012 (PAW classification)**

   The classification is based on the number of platelets, the activation of platelets, the presence or absence of white blood cells (PAW classification).
   PRP characteristics:
   Mean platelet concentration: high (P3) (platelets > 750,000 to 1,800,000 ul)
   Activation: no external activating agent was used
   White blood cells concentration: below the baseline (B)
   The final classification of the PRP used in the present study is: P3-B

3. **Magalon et al., 2016 (DEPA classification)**

   The classification considers the efficiency of production of PRP and the purity of the preparation in terms of the relative composition of platelets, white blood cells and red blood cells. Dose of administered platelets and activation are also considered (DEPA classification).
   PRP characteristics:
   Dose of injected platelets: C (1 to 3 billion). The volume of each single injection was 2 ml with a mean content of platelets of 2.36 x 10^9.
   Platelet recovery rate %: C (<30 to 70%)
   Purity of the PRP (relative composition in platelets %): B (pure PRP). Platelets are >70 to 90%.
   The final DEPA score is: CCB

Furthermore, we provide some information to meet requests of "**the minimum reporting requirements for clinical studies evaluating PRP**" as suggested by the paper by **Murray et al, 2017** entitled "Minimum Information for Studies Evaluating Biologics in Orthopaedics (MIBO): Platelet-Rich Plasma and Mesenchymal Stem Cells", Table II

**REQUIREMENT**	**description**
**Essential data regarding the clinical cases and the intervention**	Data regarding the clinical cases are reported at paragraphs 2.2, 2.4, 3.2 of the manuscript
**Whole blood processing**	• Blood collection (60ml) was performed using 5 ml of 4% trisodium citrate as anticoagulant<br>• The blood was processed within 30 minutes.<br>• Blood was stored at room temperature, in mild agitation
**PRP processing**	• PRP preparation is described at paragraph 2.3 of the manuscript<br>• Mean platelet recovery rate: 51,5%<br>• PRP was immediately used or stored at -20°C (see paragraph 2.4)
**PRP characteristics**	• Table 1 reports key data about PRP characterization.<br>• No external activating agent was used
**PRP delivery**	PRP delivery is described at paragraph 2.4
**Post operative care and outcome**	Described at paragraph 3.2, 4

BIBLIOGRAPHY

47 DeLong JM, Russell RP, Mazzocca AD. Platelet-rich plasma: the PAW classification system. Arthroscopy. 2012 Jul;28(7):998-1009. doi: 10.1016/j.arthro.2012.04.148.

24 Dohan Ehrenfest DM, Rasmusson L, Albrektsson T. Classification of platelet concentrates: from pure platelet-rich plasma (P-PRP) to leucocyte- and platelet-rich fibrin (L-PRF). Trends Biotechnol. 2009 Mar;27(3):158-67. doi: 10.1016/j.tibtech.2008.11.009.

49 Magalon J, Chateau AL, Bertrand B, Louis ML, Silvestre A, Giraudo L, Veran J, Sabatier F. DEPA classification: a proposal for standardising PRP use and a retrospective application of available devices. BMJ Open Sport Exerc Med. 2016 Feb 4;2(1):e000060. doi: 10.1136/bmjsem-2015-000060.

48 Murray IR, Geeslin AG, Goudie EB, Petrigliano FA, LaPrade RF. Minimum Information for Studies Evaluating Biologics in Orthopaedics (MIBO): Platelet-Rich Plasma and Mesenchymal Stem Cells. J Bone Joint Surg Am. 2017 May 17;99(10):809-819. doi: 10.2106/JBJS.16.00793.

46 Rossi LA, Murray IR, Chu CR, Muschler GF, Rodeo SA, Piuzzi NS. Classification systems for platelet-rich plasma. Bone Joint J. 2019;101-B(8):891-896. doi:10.1302/0301-620X.101B8.BJJ-2019-0037.R1
